# Associations of the triglyceride-glucose index and its combined indices with non-suicidal self-injury in adolescents with depressive disorders: the mediating role of sleep quality

**DOI:** 10.3389/fpsyt.2025.1689128

**Published:** 2025-12-09

**Authors:** Pu-Le Liu, Shou Gao, Yan Zhang, Jiao Li, Jing Du, Ning Yang, Qiang-Li Dong

**Affiliations:** 1 Department of Mental Health, The Second Hospital of Lanzhou University, Lanzhou, China; 2 Department of Mental Health, Mental Health Institute of Central South University, Changsha, China

**Keywords:** depressive disorder, adolescents, triglyceride-glucose index, non-suicidal self-injury, sleep quality

## Abstract

**Objective:**

This study examined the associations between the Triglyceride-Glucose (TyG) index and its combined indices (TyG-BMI, TyG-WC, TyG-WHtR) with non-suicidal self-injury (NSSI) in adolescents with Major Depressive Disorder (MDD). It also explored whether sleep quality mediated these relationships.

**Method:**

A total of 157 adolescents (12–18 years) with MDD were recruited. Participants were divided into NSSI (n = 78) and non-NSSI (n = 79) groups based on DSM-5 criteria. The TyG index and its derivatives were calculated from fasting blood samples. Analyses included multivariable logistic regression, restricted cubic spline (RCS) analysis, mediation analysis, and receiver operating characteristic (ROC) curves.

**Results:**

The NSSI group had higher TyG index values (Median [Q_1_, Q_3_]: 8.23 [7.95, 8.45]) than the non-NSSI group (7.73 [7.33, 8.21], *P* < 0.001). Adjusted regression models confirmed significant associations between TyG indices and NSSI (e.g., TyG: OR = 3.50, 95% CI: 1.82–6.74, *P* < 0.001), especially in female adolescents. Sleep quality partially mediated the link between TyG and NSSI (proportion mediated = 17.1%, *P* = 0.026). ROC analysis showed moderate predictive accuracy for TyG-WC (AUC = 0.745, 95% CI: 0.666–0.824).

**Conclusion:**

The TyG index and its combined indices are positively associated with NSSI in adolescents with MDD. Sleep quality partially mediates this relationship. These indices may serve as low-cost markers for early identification of adolescents at risk for NSSI in clinical practice.

## 1 Introduction

Major Depressive Disorder (MDD) is a prevalent mental health disorder characterized by persistent symptoms of a depressed mood (1).These symptoms usually appear during adolescence. Global studies show that about 3.7% of adolescents are affected by depressive disorders (2). At the same time, Non-Suicidal Self-Injury (NSSI) involves intentionally hurting oneself without the intention of suicide. It includes behaviors like cutting, burning, scratching, or biting the skin (3). These actions are often socially stigmatized. NSSI is seen as an unhealthy way to cope with emotions. It is closely linked to emotional problems, difficulty in relationships, and a higher risk of harmful behaviors. NSSI is also a strong predictor of suicidal thoughts and actions among adolescents (4). Studies show that NSSI is common in adolescents with depressive disorders (5). About 50% of adolescents with MDD engage in NSSI (6). Further research has shown that adolescents with both MDD and NSSI tend to have more severe symptoms. They also face worse treatment outcomes and long-term effects (7). NSSI is usually seen as a psychological or emotional response. However, more and more evidence suggest that it may not just reflect psychiatric symptoms. It could also point to problems in biological systems, especially issues in metabolic and inflammatory pathways (8).

The triglyceride-glucose (TyG) index is a recent biomarker that combines fasting triglyceride and glucose levels to assess insulin resistance (IR) (9). IR occurs when cells have difficulty absorbing insulin and using glucose, leading to higher insulin production to meet the body’s metabolic needs (10). IR is known to play a significant role in various psychiatric disorders, especially depression (11). Studies have shown that higher TyG index levels are linked to more severe depression and other psychiatric symptoms (12). The TyG index may serve as a predictor of depression severity. IR can affect brain function through inflammation, oxidative stress, and metabolic issues (13).

Emerging evidence suggests a bidirectional link between metabolic dysfunction and sleep, with some studies indicating that the TyG index may partially predict sleep disturbances. Although sleep problems can aggravate IR through neuroendocrine and inflammatory mechanisms (14–16), sleep is also strongly shaped by upstream psychosocial and lifestyle factors such as emotional state, stress, and circadian behaviors. These upstream influences subsequently affect metabolic regulation. In this framework, the TyG index reflects downstream metabolic consequences, whereas sleep serves as a modifiable behavioral mediator. Moreover, poor sleep has been associated with impaired emotion regulation, impulsivity, and an increased risk of NSSI in youth (17, 18). Therefore, sleep quality was conceptualized as the mediator in our study rather than the TyG index. The interplay among metabolic imbalance, depressive symptoms, and NSSI appears to differ across sexes (19). Existing findings suggest that adolescent girls are more susceptible to mood disorders, more sensitive to metabolic disruption (20), and more inclined toward NSSI when compared with their male counterparts. Therefore, analyses stratified by sex are essential for clarifying how these relationships diverge between boys and girls.

Previous research has explored associations between the TyG index and depressive symptoms, but its link with NSSI in depressed adolescents and its predictive value remain unclear. Sleep quality may play a central role in this pathway, as IR is often accompanied by disturbed sleep, which can exacerbate metabolic dysfunction and increase NSSI risk. Additionally, sex differences may influence these relationships, given that interactions among metabolism, sleep, and self-injurious behavior may vary between males and females. This study aims to examine the associations between the TyG index and related metabolic indicators with NSSI in adolescents with major depressive disorder. We will also investigate whether sleep quality mediates these associations and whether sex moderates these pathways.

This study aims to address the following scientific questions (1): Is the TyG index and its combined indices significantly associated with NSSI behaviors and sleep quality? (2) Does sleep quality mediate the relationship between the TyG index and its combined indices and NSSI behaviors? (3) Are these associations moderated by sex differences? and (4) Does sex influence the mediating effect of sleep quality? Accordingly, we hypothesize that (1): the TyG index and its combined indices is correlated with both NSSI behaviors and sleep quality (2); sleep quality partially mediates the relationship between the TyG index and its combined indices and NSSI behaviors; (3) the association between the TyG index and its combined indices and NSSI behaviors differs by sex; and (4) the mediating effect of sleep quality is moderated by sex.

## 2 Materials and methods

### 2.1 Study population

This study used a sequential enrollment method to select 157 adolescents, aged 12 to 18, who were diagnosed with depressive disorder and hospitalized in the Department of Mental Health at the Second Hospital of Lanzhou University between July 2022 and December 2024. The inclusion criteria were:

(1) The current episode met the diagnostic criteria for depression as per the DSM-5. (2) A score of ≥17 on the 17-item Hamilton Depression Rating Scale (HAMD-17). (3) Age between 12 and 18 years. (4) Informed consent to participate in the study, with the consent form signed. The exclusion criteria were:

(1) A history of neurological disorders, major physical illnesses, or endocrine disorders. (2) A history or current diagnosis of schizophrenia, bipolar disorder, substance use disorders, or mental disorders induced by alcohol or drugs. (3) Pregnancy or breastfeeding. (4) A direct family history of bipolar disorder or mania. (5) Modified electroconvulsive therapy (MECT) within the last 6 months. (6) Hearing impairment (assessed via a finger snap test) or color blindness.

**Figure 1:**
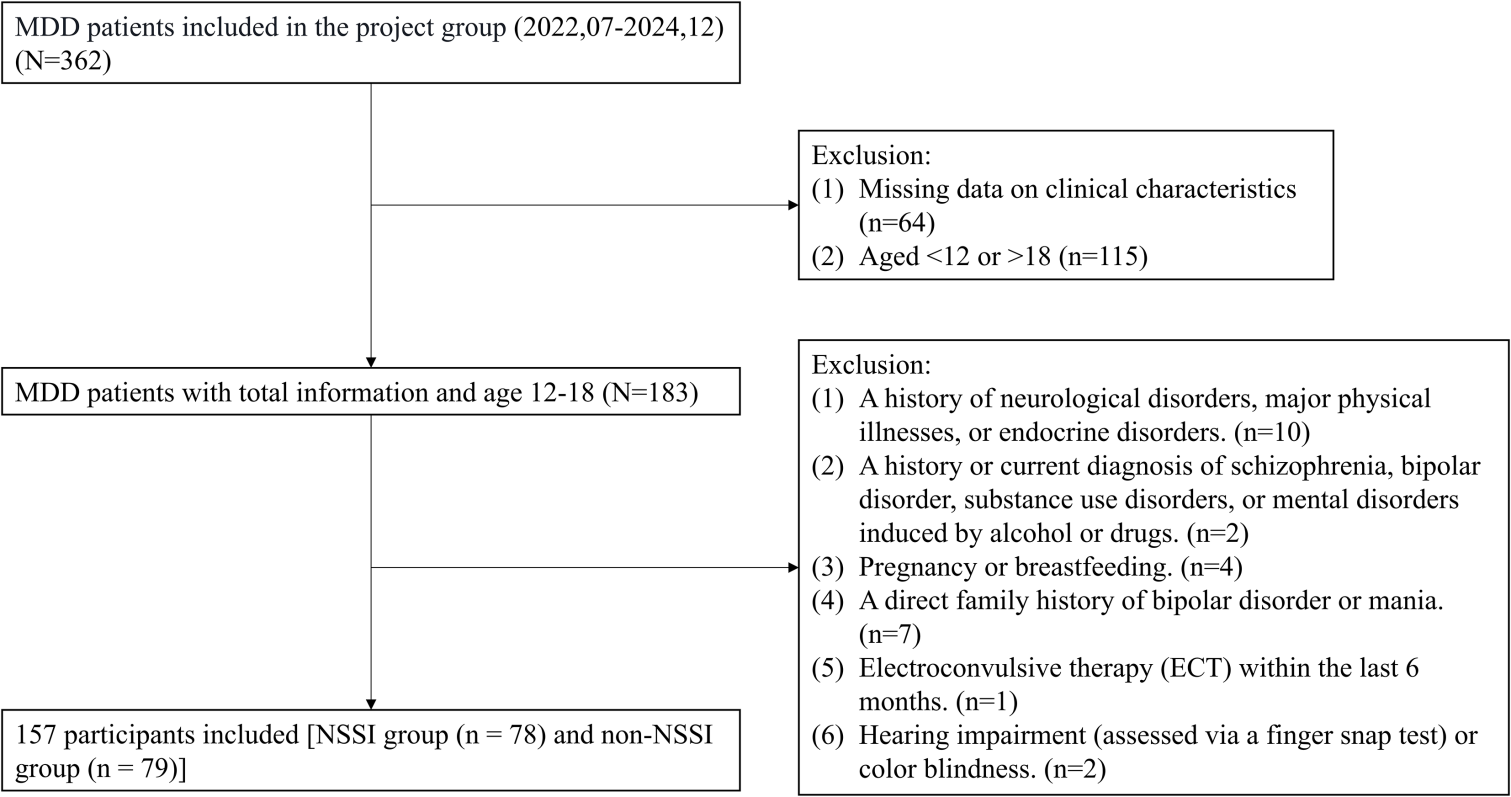
Flowchart of participant selection.

All participants were receiving selective serotonin reuptake inhibitors (SSRIs) during the study period. None of the participants were using lipid-lowering medications. Participants were divided into two groups based on the DSM-5 criteria for NSSI: the NSSI group (n = 78) and the non-NSSI group (n = 79). The study received approval from the Ethics Committee of the Second Hospital of Lanzhou University (Approval No. 2020A-134). Informed consent was obtained from all participants and their guardians. **Figure 1** shows more details about the sample, exclusion criteria, and study design.

### 2.2 Primary measures

#### 2.2.1 General information

A self-designed questionnaire was used to gather demographic data from the participants, including sex, age, per capita monthly household income (PCI), body mass index (BMI), Waist Circumference (WC), smoking, and alcohol consumption. After the participants were diagnosed by a senior psychiatrist, a specially trained psychiatrist explained the study’s content and objectives to both the participants and their guardians. The relevant assessments and sample collections were then carried out. All evaluators underwent consistency training, achieving a Kappa coefficient of 0.85.

#### 2.2.2 Assessment of NSSI

NSSI was assessed based on the criteria outlined in the DSM-5. The diagnosis was determined through a structured clinical interview conducted by trained psychiatrists or clinical psychologists.​ To ensure inter-rater consistency, all assessors participated in a standardized training session on the application of the DSM-5 criteria for NSSI. Additionally, a random sample of interviews was independently rated by a second assessor, and inter-rater reliability was calculated, showing a high level of agreement, achieving a Kappa coefficient of 0.80. The specific diagnostic criteria were as follows (21): (1) Over the past year, the individual has engaged in self-injurious behavior (e.g., cutting, scratching, burning, needle sticking, biting, hitting) on 5 or more days, with the expectation that these injuries cause mild to moderate physical harm. (2) The individual’s self-injury behavior is driven by one or more of the following: to alleviate negative feelings or cognitive states, to resolve interpersonal distress, or to create a positive emotional state. (3) The behavior is linked to at least one of the following conditions: interpersonal difficulties, negative feelings or thoughts, persistent engagement in self-injury over time, or frequent, uncontrollable thoughts of self-harm. (4) The behavior is not socially accepted (e.g., body piercings, tattoos, or part of religious or cultural rituals) and does not include behaviors like picking scabs or biting nails. (5) The behavior, or its resulting consequences, causes significant disruption in key areas such as interpersonal relationships, academic achievement, or other essential aspects of functioning. (6) The behavior does not occur during psychotic episodes, delirium, or while under the influence of substances, nor during withdrawal. In individuals with neurodevelopmental disorders, it does not form part of a repetitive or stereotyped pattern. (7) This behavior cannot be better explained by another mental health disorder or a medical condition.

#### 2.2.3 Scale assessments

Suicidal ideation was assessed using the Chinese version of the Beck Suicidal Ideation Scale (BSSI). The BSSI is commonly used to measure suicidal tendencies. The severity of suicidal ideation is assessed based on the average score of items 1 to 5. The scores, which range from 0 to 3, reflect the intensity of suicidal thoughts, with higher values signifying more pronounced ideation (22). The Cronbach’s alpha for this study sample was 0.78 (95% CI: 0.76–0.83).

Depressive severity was measured using the 17-item Hamilton Depression Rating Scale (HAMD-17). This scale is a standard tool for evaluating depressive symptoms and guiding treatment. A higher total score reflects greater severity of depression (23). The Cronbach’s alpha for this scale in our study sample was 0.88 (95% CI: 0.86–0.90).

Anxiety level was assessed using the Hamilton Anxiety Rating Scale (HAMA). This is a well-established tool for evaluating anxiety symptoms. Higher scores indicate greater anxiety severity (24). The Cronbach’s alpha for the HAMA in our study sample was 0.92 (95% CI: 0.90–0.93).

Sleep quality was assessed using the Pittsburgh Sleep Quality Index (PSQI). The scale comprises seven components (subjective sleep quality, sleep latency, sleep duration, sleep efficiency, sleep disturbances, use of sleep medication, and daytime dysfunction), with a total score ranging from 0 to 21, where higher scores indicate poorer sleep quality (25). In the present sample, the scale demonstrated good internal consistency, with a Cronbach’s alpha coefficient of 0.84 (95% CI: 0.82–0.87).

#### 2.2.4 Blood biomarker testing

Fasting venous blood samples (6 mL) were collected from all participants in the early morning. Plasma was separated by centrifugation at 3000 rpm for 15 minutes, labeled, and stored at −80 °C until analysis. A fully automated biochemical analyzer was used to quantify the following indicators: fasting plasma glucose (FPG, mg/dL), triglycerides (TG, mg/dL), total cholesterol (TC, mg/dL), high-density lipoprotein cholesterol (HDL-C, mg/dL), and low-density lipoprotein cholesterol (LDL-C, mg/dL).

The metabolic indices were calculated as follows (12): TyG = ln [TG × FPG/2]; TyG-BMI = TyG × BMI; TyG-WC = TyG × WC; TyG-WHtR = TyG × (WC/Height); METS-IR = ln (2 × FPG + TG) × BMI/ln (HDL-C); RC = TC − HDL-C − LDL-C.

BMI was calculated as weight (kg) divided by height squared (m²). WC was measured in centimeters (cm).

#### 2.2.5 Quality control

Quality control for this study was part of the baseline phase of a cohort study on adolescents with MDD, conducted by our research team. All enrolled patients had completed at least one year of outpatient follow-up and had received clear diagnoses. For patients unable to attend follow-up visits, we conducted a phone follow-up one year after baseline enrollment. This follow-up was used to update their diagnoses and exclude those who no longer met the inclusion criteria or who met any of the exclusion criteria.

### 2.3 Covariates

Continuous covariates included age. Categorical variables, used for classification, included sex (male/female), educational attainment (less than high school, high school, more than high school), per capita monthly household income (≤1250, 1251–2500, >2500 yuan), alcohol consumption (yes/used to drink/no), smoking status (yes/used to smoke/no), and the presence of comorbid conditions (yes/no). Comorbid conditions were defined as the presence of at least one of the following self-reported medical conditions: diabetes, dyslipidemia, kidney failure, kidney stones, heart failure, stroke, liver disease, rheumatoid arthritis, or cancer.

### 2.4 Statistical analysis

All statistical procedures were performed in R software, version 4.5.1 (https://cran.r-project.org/), employing specialized packages for interaction modeling. A two-sided p-value of less than 0.05 was regarded as the threshold for statistical significance. Normally distributed continuous data are reported as Mean ± Standard Deviation (SD), and differences between two groups were assessed using the independent-samples t-test. Variables with skewed distributions are expressed as Median (Q_1_, Q_3_), with group comparisons conducted via the Mann–Whitney rank-sum test. Categorical data are presented as frequencies and percentages [n (%)], and comparisons were made using the chi-square test or, when assumptions were not met, Fisher’s exact test.

To evaluate associations among the TyG index, its derived indicators, sleep quality, and NSSI, we applied multivariable logistic regression as well as generalized linear models (GLM). To identify potential sex-related differences, all analyses were stratified by male and female participants. Furthermore, mediation analysis was conducted to determine whether depressive symptoms served as an intermediary variable in the relationship between the TyG index and NSSI. This mediation framework utilized 1,000 bootstrap resamples to estimate indirect effects, quantify the proportion mediated, and establish significance levels. Finally, the receiver operating characteristic (ROC) curves were generated from multivariable logistic regression models adjusted for age, sex, BMI, and other relevant covariates. Each metabolic index (TyG, TyG-BMI, TyG-WC, TyG-WHtR) was included separately as the single predictor in the model. The area under the curve (AUC) and 95% confidence interval (CI) were calculated to assess discriminatory ability.

## 3 Results

### 3.1 Participant characteristics

A total of 157 adolescents with MDD were included, of whom 78 (49.68%) reported NSSI, and 79 (50.32%) did not. Regarding lifestyle factors, the NSSI group had higher rates of alcohol use (29.49% vs. 10.13%, *P* = 0.004) and smoking (20.51% vs. 6.33%, *P* = 0.015). Clinically, adolescents with NSSI showed more severe depressive symptoms (HAMD: 22.85 ± 5.31 vs. 19.32 ± 7.21, *P* < 0.001), anxiety symptoms (HAMA: 27.30 ± 7.01 vs. 24.19 ± 9.57, *P* = 0.021), and suicidal ideation (BSSI: 2.37 ± 0.54 vs. 1.82 ± 0.79, *P* < 0.001), as well as poorer sleep quality (PSQI: 14.17 ± 4.13 vs. 9.44 ± 5.18, *P* < 0.001).

Metabolic measurements revealed that the NSSI group had higher waist circumference (WC: 69.12 ± 4.42 vs. 66.40 ± 4.17, *P* < 0.001), waist-to-height ratio (WHtR: 41.41 ± 3.06 vs. 40.19 ± 3.09, *P* = 0.014), total cholesterol, triglycerides, and LDL-C levels. Moreover, the NSSI group exhibited significantly higher TyG index and its combined indices (TyG-BMI, TyG-WC, TyG-WHtR; all *P* < 0.01). Detailed results are presented in **Table 1**.

**Table 1:** Comparison of variables between non-NSSI and NSSI groups.

Variables	Total (n = 157)	Non-NSSI (n = 79)	NSSI (n = 78)	Statistic	*P*
Age (year), Mean ± SD	15.36 ± 1.77	15.29 ± 1.59	15.44 ± 1.94	t=-0.51	0.610
Gender, n (%)				χ²=4.48	0.034
Male	55 (35.03)	34 (43.04)	21 (26.92)		
Female	102 (64.97)	45 (56.96)	57 (73.08)		
Education, n (%)				χ²=0.41	0.813
< High School	67 (42.68)	35 (44.30)	32 (41.03)		
High School	76 (48.41)	38 (48.10)	38 (48.72)		
> High School	14 (8.92)	6 (7.59)	8 (10.26)		
PCI (yuan), n (%)				χ²=0.10	0.950
≤1250	46 (29.30)	23 (29.11)	23 (29.49)		
1251 – 2500	69 (43.95)	34 (43.04)	35 (44.87)		
>2500	42 (26.75)	22 (27.85)	20 (25.64)		
Comorbid, n (%)				χ²=2.18	0.140
Yes	36 (22.93)	22 (27.85)	14 (17.95)		
No	121 (77.07)	57 (72.15)	64 (82.05)		
Alcohol, n (%)				-	0.004
Yes	31 (19.75)	8 (10.13)	23 (29.49)		
Used to Drink	2 (1.27)	1 (1.27)	1 (1.28)		
No	124 (78.98)	70 (88.61)	54 (69.23)		
Smoke, n (%)				-	0.015
Yes	21 (13.38)	5 (6.33)	16 (20.51)		
Used to Smoke	4 (2.55)	3 (3.80)	1 (1.28)		
No	132 (84.08)	71 (89.87)	61 (78.21)		
Frequency (year times), Mean ± SD	1.73 ± 2.07	0.00 ± 0.00	3.47 ± 1.59	t=-19.36	<0.001
Frequency (month times), Mean ± SD	6.96 ± 7.85	0.89 ± 1.38	13.12 ± 6.84	t=-15.48	<0.001
Self-harm Methods, n (%)				-	<0.001
Never	47 (29.94)	47 (59.49)	0 (0.00)		
Cutting	83 (52.87)	25 (31.65)	58 (74.36)		
Scratching	6 (3.82)	2 (2.53)	4 (5.13)		
Burning	11 (7.01)	3 (3.80)	8 (10.26)		
Needle Sticking	5 (3.18)	1 (1.27)	4 (5.13)		
Biting	3 (1.91)	1 (1.27)	2 (2.56)		
Hitting	2 (1.27)	0 (0.00)	2 (2.56)		
HAMD, Mean ± SD	21.07 ± 6.56	19.32 ± 7.21	22.85 ± 5.31	t=-3.50	**<0.001**
HAMA, Mean ± SD	25.74 ± 8.51	24.19 ± 9.57	27.30 ± 7.01	t=-2.33	**0.021**
BSSI, Mean ± SD	2.10 ± 0.73	1.82 ± 0.79	2.37 ± 0.54	t=-5.07	**<0.001**
DSST, Mean ± SD	52.66 ± 12.37	53.56 ± 11.74	51.76 ± 13.00	t=0.91	0.364
PSQI, Mean ± SD	11.79 ± 5.24	9.44 ± 5.18	14.17 ± 4.13	t=-6.32	**<0.001**
Height, Mean ± SD	1.66 ± 0.08	1.66 ± 0.09	1.67 ± 0.08	t=-1.17	0.245
Weight, Mean ± SD	55.78 ± 11.21	54.32 ± 10.88	57.27 ± 11.41	t=-1.66	0.099
WC, Mean ± SD	67.75 ± 4.49	66.40 ± 4.17	69.12 ± 4.42	t=-3.97	**<0.001**
BMI, Mean ± SD	20.11 ± 3.67	19.75 ± 3.39	20.47 ± 3.93	t=-1.22	0.223
WHtR, Mean ± SD	40.79 ± 3.13	40.19 ± 3.09	41.41 ± 3.06	t=-2.49	**0.014**
FPG (mg/dL), M (Q_1_, Q_3_)	83.88 (78.84, 88.02)	83.34 (78.93, 87.39)	84.51 (78.97, 88.96)	Z=-0.90	0.368
TC (mg/dL), M (Q_1_, Q_3_)	131.48 (108.28, 150.43)	109.82 (96.67, 131.48)	147.91 (130.03, 169.18)	Z=-7.06	**<0.001**
TG (mg/dL), M (Q_1_, Q_3_)	73.51 (46.06, 106.28)	50.48 (38.53, 84.14)	86.80 (65.76, 113.59)	Z=-4.34	**<0.001**
HDL-C (mg/dL), M (Q_1_, Q_3_)	42.92 (38.67, 49.11)	43.70 (39.25, 48.72)	42.54 (33.74, 49.40)	Z=-0.48	0.628
LDL-C (mg/dL), M (Q_1_, Q_3_)	78.50 (61.49, 97.45)	63.03 (52.78, 75.02)	96.09 (84.40, 110.31)	Z=-8.65	**<0.001**
TyG, M (Q_1_, Q_3_)	8.07 (7.53, 8.39)	7.73 (7.33, 8.21)	8.23 (7.95, 8.45)	Z=-4.37	**<0.001**
TyG-BMI, M (Q_1_, Q_3_)	153.74 (140.42, 176.82)	146.77 (134.39, 166.85)	159.32 (145.55, 182.18)	Z=-2.68	**0.007**
TyG-WC, M (Q_1_, Q_3_)	536.37 (502.45, 573.84)	509.57 (471.47, 545.10)	559.02 (530.38, 588.22)	Z=-5.30	**<0.001**
TyG-WHtR, M (Q_1_, Q_3_)	322.18 (296.76, 347.48)	302.70 (284.21, 328.58)	333.51 (317.21, 355.18)	Z=-4.78	**<0.001**
METS-IR, M (Q_1_, Q_3_)	28.16 (25.63, 32.58)	27.61 (25.32, 32.16)	28.77 (26.04, 32.69)	Z=-1.37	0.171
RC, M (Q_1_, Q_3_)	8.12 (3.87, 13.92)	8.12 (3.29, 13.92)	8.31 (5.12, 15.47)	Z=-1.11	0.269

t: t-test, Z: Mann-Whitney test, χ²: Chi-square test, -: Fisher exact.

SD: standard deviation, M: Median, Q_1_: 1st Quartile, Q_3_: 3st Quartile.

PCI, Per Capita Income; HAMD, Hamilton Depression Rating Scale; HAMA, Hamilton Anxiety Rating Scale; BSSI, Beck Scale for Suicide Ideation; DSST, Digit Symbol Substitution Test; PSQI, Pittsburgh Sleep Quality Index; WC, Waist Circumference; BMI, Body Mass Index; WHtR, Waist-to-Height Ratio; FPG, Fasting Plasma Glucose; TC, Total Cholesterol; TG, Triglyceride; HDL-C, High-Density Lipoprotein Cholesterol; LDL-C, Low-Density Lipoprotein Cholesterol; TyG, Triglyceride-Glucose Index; TyG-BMI, Triglyceride-Glucose Index with Body Mass Index; TyG-WC, Triglyceride-Glucose Index with Waist Circumference; TyG-WHtR, Triglyceride-Glucose Index with Waist-to-Height Ratio; METS-IR, Metabolic Score for Insulin Resistance; RC, Remnant Cholesterol.

Bold values denote a statistically significant difference (P < 0.05).

### 3.2 Correlations between TyG index and NSSI

**Figure 2:**
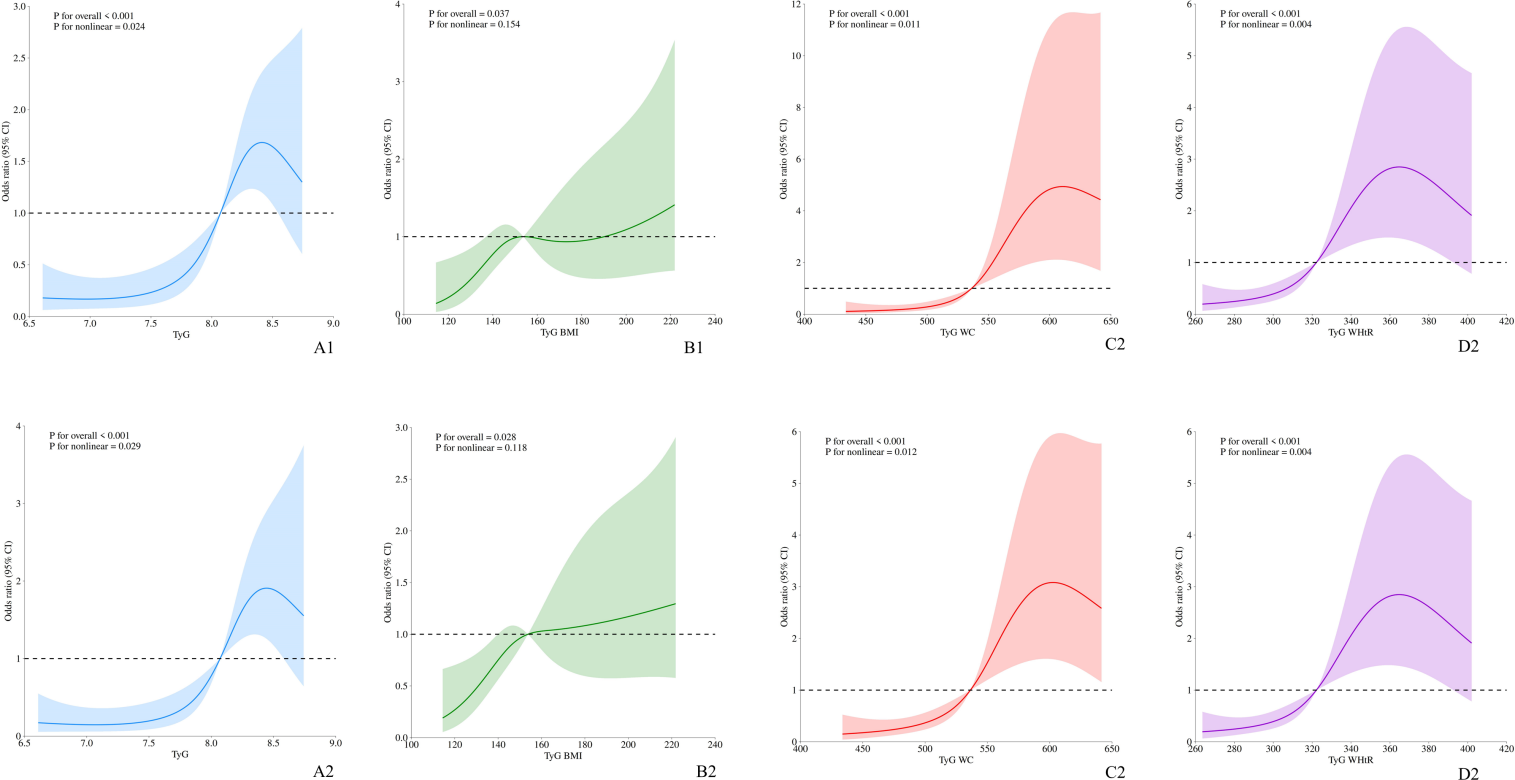
**(A)** The association between the TyG index and NSSI; **(B)** The association between the TyG-BMI index and NSSI; **(C)** The association between the TyG-WC index and NSSI; **(D)** The association between the TyG-WHtR index and NSSI. Model1: Crude; Model2: Adjusted: Age, Sex, Alcohol, Smoke, Education, Comorbid, PCI.

After adjusting for age, sex, alcohol use, smoking, education, comorbidity, and PCI, higher TyG index and its combined indices (TyG-BMI, TyG-WC, TyG-WHtR) were significantly associated with increased risk of NSSI and poorer sleep quality (all *P* < 0.01). Restricted cubic spline analyses revealed that the associations were predominantly nonlinear, exhibiting J-shaped or monotonically increasing trends (**Figure 2**). Notably, the risks of NSSI and poor sleep quality increased more sharply after the TyG index and its combined indices exceeded certain thresholds. In the total sample, the TyG index remained a strong predictor of NSSI (adjusted OR = 3.50, 95% CI: 1.82–6.74, *P* < 0.001) and sleep quality (β = 3.56, 95% CI: 2.43–4.68, *P* < 0.001). Stratified analyses showed stronger associations among females, with adjusted OR for NSSI reaching 7.69 (95% CI: 2.67–22.11, *P* < 0.001), whereas associations in males were weaker and not statistically significant. Similarly, TyG-related indices were consistently associated with poor sleep quality in both sexes, but the effect sizes were larger in females.

Moreover, NSSI itself was significantly correlated with poor sleep quality (adjusted OR = 1.27, 95% CI: 1.15–1.40, *P* < 0.001). Detailed results are presented in **Table 2**; **Figure 2**.

**Table 2:** Associations between TyG/TyG-BMI/TyG-WC/TyG-WHtR index, NSSI and sleep quality.

Variables	Crude β/OR (95%CI)	*P*-value	Adjusted β/OR (95%CI)	*P*-value
Associations of TyG index with NSSI	Crude OR (95%CI)	*P*-value	Adjusted OR (95%CI)	*P*-value
Total	3.1107 (1.7320–5.5800)	<0.001	3.5007 (1.8175–6.7403)	<0.001
Male	1.7490 (0.7262, 4.2139)	0.2120	2.1656 (0.7289, 6.4316)	0.1642
Female	5.1261 (2.2223, 11.8235)	<0.001	7.6910 (2.6731, 22.1110)	<0.001
Associations of TyG index with Sleep quality	Crude β (95%CI)	*P*-value	Adjusted β (95%CI)	*P*-value
Total	3.5659 (2.4750, 4.6568)	<0.001	3.5554 (2.4335, 4.6773)	<0.001
Male	2.5228 (0.7892, 4.2564)	<0.001	2.9273 (1.2235, 4.6311)	0.0015
Female	4.1121 (2.7148, 5.5094)	<0.001	3.9969 (2.5332, 5.4606)	<0.001
Associations of TyG-BMI with NSSI	Crude OR (95%CI)	*P*-value	Adjusted OR (95%CI)	*P*-value
Total	1.0125 (1.0025–1.0227)	<0.001	1.0135 (1.0026–1.0246)	<0.001
Male	0.9978 (0.9809–1.0150)	0.8000	1.0026 (1.0015, 1.0038)	<0.001
Female	1.0225 (1.0069–1.0384)	0.0046	1.0246 (1.0076–1.0420)	0.0043
Associations of TyG-BMI with Sleep quality	Crude β (95%CI)	*P*-value	Adjusted β (95%CI)	*P*-value
Total	0.0378 (0.0155–0.0600)	0.0011	0.0412 (0.0184–0.0641)	<0.001
Male	0.0286 (−0.0092–0.0664)	0.144	0.0334 (−0.0041–0.0708)	0.087
Female	0.0419 (0.0142–0.069)	0.0038	0.0440 (0.0142–0.0738)	0.0047
Associations of TyG-WC with NSSI	Crude OR (95%CI)	*P*-value	Adjusted OR (95%CI)	*P*-value
Total	1.0143 (1.0078–1.0208)	<0.001	1.0180 (1.0101–1.0259)	<0.001
Male	1.0078 (0.9983–1.0173)	0.1074	1.0087 (0.9976–1.0200)	0.1270
Female	1.0255 (1.0140–1.0371)	<0.001	1.0320 (1.0169–1.0473)	<0.001
Associations of TyG-WC with Sleep quality	Crude β (95%CI)	*P*-value	Adjusted β (95%CI)	*P*-value
Total	0.0336 (0.0224–0.0447)	<0.001	0.0348 (0.0232–0.0464)	<0.001
Male	0.0263 (0.0077–0.0449)	0.0076	0.0294 (0.0113–0.0475)	0.0026
Female	0.0371 (0.0230–0.0513)	<0.001	0.0381 (0.0229–0.0532)	<0.001
Associations of TyG-WHtR with NSSI	Crude OR (95%CI)	*P*-value	Adjusted OR (95%CI)	*P*-value
Total	1.0182 (1.0088–1.0278)	<0.001	1.0194 (1.0092–1.0296)	<0.001
Male	1.0039 (0.9912–1.0168)	0.5460	1.0069 (0.9913–1.0227)	0.3887
Female	1.0305 (1.0153–1.0460)	<0.001	1.0367 (1.0182–1.0555)	<0.001
Associations of TyG-WHtR with Sleep quality	Crude β (95%CI)	*P*-value	Adjusted β (95%CI)	*P*-value
Total	0.0471 (0.0291–0.0651)	<0.001	0.0486 (0.0303–0.0669)	<0.001
Male	0.0360 (0.0085–0.0634)	0.0131	0.0391 (0.0113–0.0669)	0.0083
Female	0.0549 (0.0313–0.0786)	<0.001	0.0558 (0.0311–0.0804)	<0.001
Associations of NSSI and Sleep quality	Crude OR (95%CI)	*P*-value	Adjusted OR (95%CI)	*P*-value
Total	1.2519 (1.1456 – 1.3663)	<0.001	1.2713 (1.1528 – 1.4027)	<0.001
Male	1.2907 (1.0779 – 1.5441)	0.0053	1.4305 (1.1049 – 1.8520)	0.0065
Female	1.2513 (1.1303 – 1.3846)	<0.001	1.2874 (1.1399 – 1.4540)	<0.001

CI, Confidence Interval.

Model1: Crude.

Model2: Adjusted: Age, Sex, Alcohol, Smoke, Education, Comorbid, PCI.

### 3.3 Mediating role of sleep quality in the relationship between TyG index and NSSI

Mediation analysis revealed that the TyG index significantly mediated the relationship between sleep quality and NSSI in adolescents with MDD. ACME represents the average causal mediation effects (i.e., the indirect effect), while ADE refers to the average direct effect, and PM refers to the proportion of the total effect that is mediated by sleep quality. In the total sample, the adjusted ACME was 0.0027 (95% CI: 0.00014–0.00942, *P* = 0.026), accounting for 17.1% of the total effect (Total effect: β = 0.0156, 95% CI: 0.0040, 0.0225). The direct effect (ADE) remained significant (β = 0.0130, 95% CI: 0.0034–0.0187, *P* < 0.001), indicating partial mediation.

**Figure 3:**
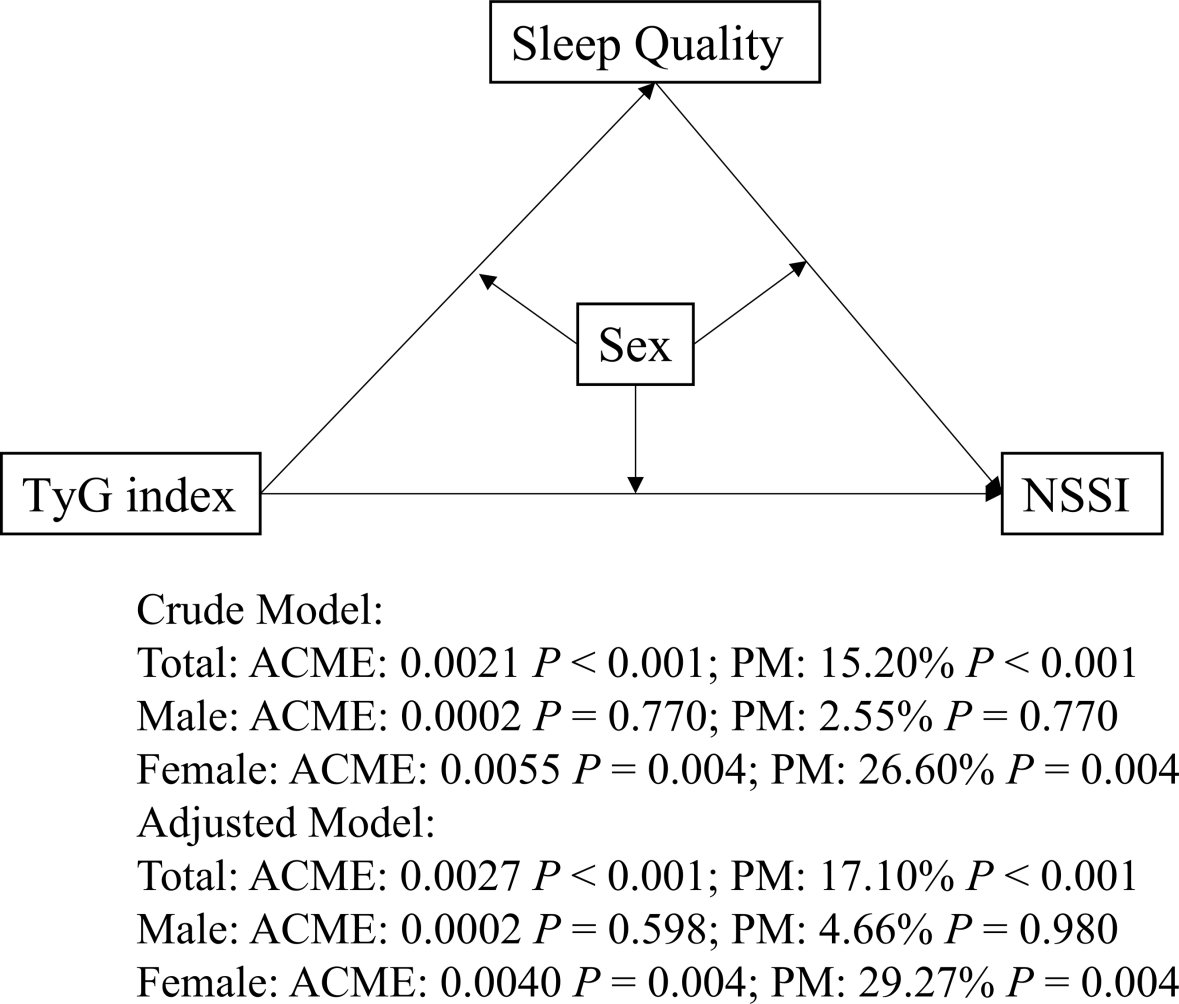
Mediation Models. The figure illustrates mediation models with TyG index as the independent variable, sleep quality as the mediators, and NSSI as the dependent variable. ACME, average causal mediation effects; ADE, average direct effect; PM, proportion mediated.

Sex-stratified analyses showed a significant mediation effect in females (adjusted ACME = 0.0040, 95% CI: 0.0002–0.0136, *P* = 0.004; proportion mediated = 29.3%), whereas no significant mediation was observed in males. Detailed results are presented in **Table 3**; **Figure 3**.

**Table 3:** Mediating role of sleep quality in the relationship between TyG index and NSSI.

Variables	Crude β	95%CI (Lower, Upper)	*P*-value	Adjusted β	95%CI (Lower, Upper)	*P*-value
ACME						
Total	0.0021	0.0001, 0.0079	0.048	0.0027	0.00014, 0.00942	0.026
Male	0.0002	-0.0011, 0.0056	0.770	0.0002	-0.0026, 0.0069	0.598
Female	0.0055	0.0004, 0.0165	0.004	0.0040	0.0002, 0.0136	0.004
ADE						
Total	0.0118	0.0027, 0.0182	<0.001	0.0130	0.0034, 0.0187	<0.001
Male	0.0063	0.0001, 0.0156	0.006	0.0048	0.0001, 0.0166	0.048
Female	0.0152	0.0029, 0.0211	0.008	0.0098	0.0009, 0.0159	0.008
Total Effect						
Total	0.0139	0.0030, 0.0220	<0.001	0.0156	0.0040, 0.0225	<0.001
Male	0.0065	0.0001, 0.0168	<0.001	0.0050	0.0001, 0.0176	0.024
Female	0.0206	0.0041, 0.0287	<0.001	0.0139	0.0013, 0.0230	<0.001
Prop. Mediated						
Total	0.1520	0.0017, 0.4244	0.048	0.1710	0.0201, 0.4771	0.026
Male	0.0255	-0.1503, 0.4555	0.770	0.0466	-0.6406, 0.7022	0.980
Female	0.2660	0.0625, 0.7293	0.004	0.2927	0.0839, 0.7832	0.004

Model1: Crude.

Model2: Adjusted: Age, Sex, Alcohol, Smoke, Education, Comorbid, PCI.

Abbreviations: ACME, average causal mediation effects; ADE, average direct effect; PM, proportion mediated.

### 3.4 ROC curve analysis

**Figure 4:**
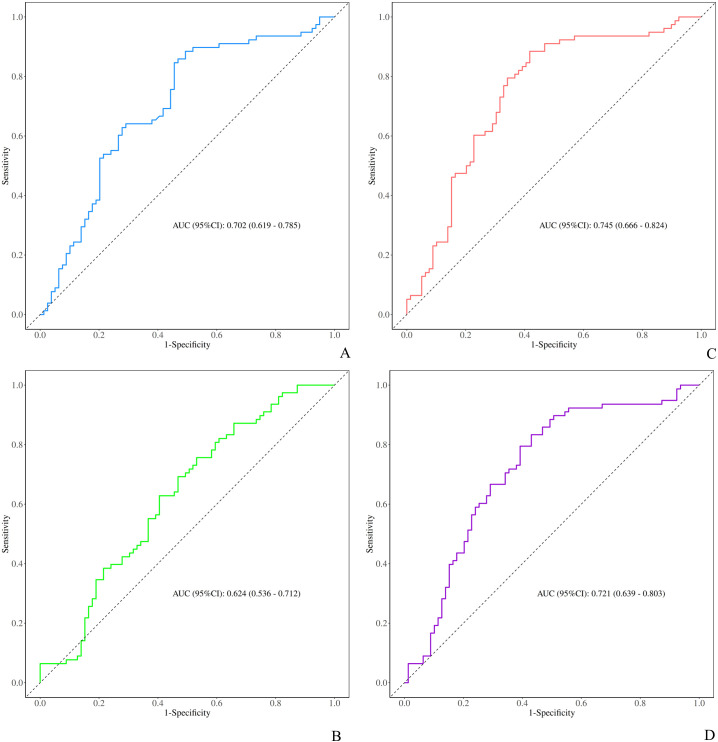
ROC curve analysis of the diagnostic efficacy. **(A)** TyG, **(B)** TyG-BMI, **(C)** TyG-WC, and **(D)** TyG-WHtR for NSSI.

ROC curves derived from the covariate-adjusted logistic regression models demonstrated that all four indices had moderate discriminatory ability for identifying adolescents with NSSI. The curve plots the relationship between the false positive rate (FPR) and the true positive rate (TPR), with the area under the curve (AUC) serving as the measure of discriminatory performance. The AUC (95% CI) values were as follows: TyG index, 0.702 (0.619–0.785); TyG-BMI, 0.624 (0.536–0.712); TyG-WC, 0.745 (0.666–0.824); and TyG-WHtR, 0.721 (0.639–0.803). Detailed results are presented in **Figure 4**.

## 4 Discussion

This study investigated the associations among the TyG index, its derived indicators, sleep quality, and NSSI in a sample of 157 adolescents diagnosed with depressive disorder. Participants were enrolled from the Department of Mental Health at the Second Hospital of Lanzhou University between July 2022 and December 2024. Our principal findings revealed significant, often nonlinear (J-shaped) relationships between the TyG index-related metrics and the risks of NSSI and poor sleep, with these associations being substantially stronger in female participants. Importantly, sleep quality was identified as a significant mediator in the TyG-NSSI pathway. The TyG index, a cost-effective and reliable surrogate marker of IR that overcomes the limitations of more complex measures like HEC and HOMA-IR (26–28), may thus serve as a valuable tool for identifying adolescents with depression at high risk for NSSI.

The robust association between the TyG index and NSSI suggests that IR is a key underlying biological pathway. While the exact mechanism requires further elucidation, IR likely contributes to NSSI risk through multiple interconnected pathways. First, IR is linked to disturbances in brain neurochemistry, including impaired dopamine function and increased monoamine oxidase (MAO) activity, which can lead to emotional dysregulation and depressive behaviors—core features of NSSI (29–32). Second, the chronic inflammation often accompanying IR (33) can directly impact brain circuits responsible for emotional control and impulse regulation (34), potentially lowering the threshold for self-injurious behaviors as a maladaptive coping strategy. The observed J-shaped relationship further suggests a critical tipping point, beyond which the physiological burden of IR accelerates neuropsychiatric risks, highlighting the potential for targeted interventions in individuals exceeding this threshold.

Mediation analyses showed that sleep quality partially explained the link between the TyG index and NSSI in adolescents with depression. Higher TyG index values were associated with poorer sleep. Poor sleep, in turn, was linked to a higher risk of NSSI. This suggests that disrupted sleep may be an important pathway through which metabolic problems contribute to self-harming behaviors. Mechanistically, IR and high triglycerides, reflected by elevated TyG index values, may disturb circadian rhythms and change the secretion of sleep-related hormones like melatonin and cortisol (35, 36). Poor sleep can further affect emotional regulation by altering prefrontal-limbic brain circuits (37). This can increase impulsivity, amplify negative emotions, and reduce stress coping. In adolescents, these effects may increase vulnerability to self-injury as a way to handle emotional distress. These findings match previous research showing that poor or insufficient sleep can worsen emotional dysregulation, impulsivity, and negative mood, all known risk factors for NSSI. Identifying sleep quality as a mediating factor suggests that improving sleep may reduce the impact of metabolic disturbances on self-injury risk in adolescents with higher TyG index levels.

The associations between the TyG index, its derived measures, and NSSI were notably stronger in female participants. Sex-stratified analyses revealed that females with elevated TyG index values experienced poorer sleep and a greater likelihood of NSSI compared to males with similar metabolic profiles. These findings align with a growing body of evidence indicating that adolescent girls are more vulnerable to the psychopathological consequences of metabolic disturbances. For instance, previous studies have reported that the relationship between obesity, insulin resistance, and depression is more pronounced in females than in males (38, 39). Our study extends this literature by demonstrating that this sex-specific pattern also applies to the risk of NSSI, with sleep quality acting as a significant mediating pathway predominantly in females. Several mechanisms may underpin this sex disparity. Females typically exhibit greater hormonal fluctuations (e.g., estrogen and progesterone), which can modulate insulin sensitivity and emotional regulation circuits (40). Additionally, sex differences in stress response systems (e.g., the HPA axis) and neural circuits governing emotion and impulse control may render females more susceptible to the interplay between metabolic dysregulation and negative emotional states (41). Crucially, our study adds a novel dimension to existing evidence by not only confirming the sex difference in the TyG–NSSI association but also quantitatively revealing a strong, significant mediating effect of sleep quality in females that was absent in males. These findings underscore the necessity of considering sex-specific pathways, such as sleep disruption, when assessing metabolic risk for self-injurious behaviors and developing targeted prevention strategies in clinical practice.

This study is the first to explore the link between the TyG index and NSSI in adolescents with depression. The TyG index is a simple and cost-effective tool that can be used in clinical settings. Notably, our comparison of various TyG-related indices revealed that TyG-WC had the highest predictive accuracy for NSSI.​ This suggests that a marker combining insulin resistance and central obesity is more strongly associated with NSSI risk than insulin resistance alone. The TyG-WC index is clinically practical and may be the most useful tool among those evaluated for risk stratification, highlighting the importance of assessing central adiposity in the metabolic management of adolescents with depression.

However, there are a few important limitations. The study used a cross-sectional design, so it cannot prove causality. While our results show a significant link between the TyG index and NSSI in adolescents with depression, we cannot determine which comes first. Cross-sectional studies only collect data at one point in time, making it impossible to establish cause and effect. We found higher TyG index values in adolescents with depression and NSSI. However, it is also possible that the TyG index contributed to the development of depression and NSSI, rather than the other way around. TyG index, sleep quality, and NSSI. Second, the relatively small sample size, particularly when further reduced in subgroup analyses (e.g., stratification by sex), may limit the statistical power and increase the risk of chance findings. Additionally, the study participants were all hospitalized patients. We did not include healthy controls, which limits the generalizability of the findings. The results may not apply to other groups of adolescents with depression. Although we considered various factors, genetic factors and early life experiences could still play a major role in the development of NSSI. For example, genetic differences might make some people more likely to experience both NSSI and depression (42). This makes it difficult to separate their effects. Also, experiences like childhood trauma or living in a disadvantaged situation can affect both mental and physical health (43). These factors can make it harder to fully understand the relationship between the TyG index and NSSI. Future research should conduct longitudinal studies to better understand the cause-and-effect relationship between the TyG index and NSSI. Expanding the study population to include more diverse groups of adolescents would help make the findings more applicable to a wider range of people. Researchers should also focus on the role of genetic and psychosocial factors in these associations. These factors may have a significant impact on the relationship between the TyG index and NSSI.

## 5 Conclusion

This study shows a significant relationship between the TyG index, its derived indicators, sleep quality, and NSSI in adolescents with depressive disorder. The associations were nonlinear, with higher TyG-related measures linked to a greater risk of NSSI, especially in female participants. Sleep quality partly mediated this relationship, suggesting it may be an important target for prevention and treatment. Among the measures studied, TyG-WC had the strongest ability to identify adolescents at higher risk of NSSI. These results indicate that the TyG index and its derivatives could be useful, low-cost tools for early screening and targeted prevention of self-injurious behaviors in clinical practice.

## Data Availability

The raw data supporting the conclusions of this article will be made available by the authors, without undue reservation.
